# Supplementary material for: Resolving the full spectrum of human genome variation using Linked-Reads
Source: Genome Res. 2019 Apr;29(4):635–45. doi: 10.1101/gr.234443.118 (PMC6442396; doi:10.1101/gr.234443.118)
Supplement: Supplemental Material [file supp_gr.234443.118_Supplemental_longranger-2.2.zip › longranger-2.2.tar.bz2!/lr2.2-public/lib/stan/cmdstan/stan/doc/api/html/agrad_2fwd_2matrix_2dot__product_8hpp_source.html]

Stan: src/stan/agrad/fwd/matrix/dot\_product.hpp Source File


|  |  |
| --- | --- |
|  | Stan  2.5.0  probability, sampling & optimization |


- Main Page
- Related Pages
- Namespaces
- Classes
- Files

- File List
- File Members

All Classes Namespaces Files Functions Variables Typedefs Enumerations Enumerator Friends Macros Pages

- src
- stan
- agrad
- fwd
- matrix

dot\_product.hpp

Go to the documentation of this file.

1 #ifndef STAN\_\_AGRAD\_\_FWD\_\_MATRIX\_\_DOT\_PRODUCT\_HPP

2 #define STAN\_\_AGRAD\_\_FWD\_\_MATRIX\_\_DOT\_PRODUCT\_HPP

3

4 #include <vector>

5 #include <stan/math/matrix/Eigen.hpp>

6 #include <stan/math/matrix/typedefs.hpp>

7 #include <stan/math/error\_handling/matrix/check\_vector.hpp>

8 #include <stan/math/error\_handling/matrix/check\_matching\_sizes.hpp>

9 #include <stan/agrad/fwd.hpp>

10 #include <stan/agrad/fwd/matrix/typedefs.hpp>

11 #include <stan/agrad/fwd/matrix/to\_fvar.hpp>

12

13 namespace stan {

14  namespace agrad {

15

16  //dot\_product for vec (in matrix) \* vec (in matrix); does all combos of row row, col col, row col, col row

17  template<typename T, int R1, int C1, int R2, int C2>

18  inline

19  fvar<T>

20  dot\_product(const Eigen::Matrix<fvar<T>, R1, C1>& v1,

21  const Eigen::Matrix<fvar<T>, R2, C2>& v2) {

22  stan::math::check\_vector("dot\_product(%1%)",v1,"v1",(double\*)0);

23  stan::math::check\_vector("dot\_product(%1%)",v2,"v2",(double\*)0);

24  stan::math::check\_matching\_sizes("dot\_product(%1%)",v1,"v1",

25  v2,"v2",(double\*)0);

26

27  fvar<T> ret(0,0);

28  for(size\_type i = 0; i < v1.size(); i++)

29  ret += v1(i) \* v2(i);

30  return ret;

31  }

32

33  template<typename T, int R1, int C1, int R2, int C2>

34  inline

35  fvar<T>

36  dot\_product(const Eigen::Matrix<fvar<T>, R1, C1>& v1,

37  const Eigen::Matrix<double, R2, C2>& v2) {

38  stan::math::check\_vector("dot\_product(%1%)",v1,"v1",(double\*)0);

39  stan::math::check\_vector("dot\_product(%1%)",v2,"v2",(double\*)0);

40  stan::math::check\_matching\_sizes("dot\_product(%1%)",v1,"v1",

41  v2,"v2",(double\*)0);

42

43  fvar<T> ret(0,0);

44  for(size\_type i = 0; i < v1.size(); i++)

45  ret += v1(i) \* v2(i);

46  return ret;

47  }

48

49  template<typename T, int R1, int C1, int R2, int C2>

50  inline

51  fvar<T>

52  dot\_product(const Eigen::Matrix<double, R1, C1>& v1,

53  const Eigen::Matrix<fvar<T>, R2, C2>& v2) {

54  stan::math::check\_vector("dot\_product(%1%)",v1,"v1",(double\*)0);

55  stan::math::check\_vector("dot\_product(%1%)",v2,"v2",(double\*)0);

56  stan::math::check\_matching\_sizes("dot\_product(%1%)",v1,"v1",

57  v2,"v2",(double\*)0);

58

59  fvar<T> ret(0,0);

60  for(size\_type i = 0; i < v1.size(); i++)

61  ret += v1(i) \* v2(i);

62  return ret;

63  }

64

65  template<typename T, int R1,int C1, int R2, int C2>

66  inline

67  fvar<T>

68  dot\_product(const Eigen::Matrix<fvar<T>, R1, C1>& v1,

69  const Eigen::Matrix<fvar<T>, R2, C2>& v2,

70  size\_type& length) {

71  stan::math::check\_vector("dot\_product(%1%)",v1,"v1",(double\*)0);

72  stan::math::check\_vector("dot\_product(%1%)",v2,"v2",(double\*)0);

73

74  fvar<T> ret(0,0);

75  for(size\_type i = 0; i < length; i++)

76  ret += v1(i) \* v2(i);

77  return ret;

78  }

79

80  template<typename T, int R1,int C1, int R2, int C2>

81  inline

82  fvar<T>

83  dot\_product(const Eigen::Matrix<fvar<T>, R1, C1>& v1,

84  const Eigen::Matrix<double, R2, C2>& v2,

85  size\_type& length) {

86  stan::math::check\_vector("dot\_product(%1%)",v1,"v1",(double\*)0);

87  stan::math::check\_vector("dot\_product(%1%)",v2,"v2",(double\*)0);

88

89  fvar<T> ret(0,0);

90  for(size\_type i = 0; i < length; i++)

91  ret += v1(i) \* v2(i);

92  return ret;

93  }

94

95  template<typename T, int R1,int C1, int R2, int C2>

96  inline

97  fvar<T>

98  dot\_product(const Eigen::Matrix<double, R1, C1>& v1,

99  const Eigen::Matrix<fvar<T>, R2, C2>& v2,

100  size\_type& length) {

101  stan::math::check\_vector("dot\_product(%1%)",v1,"v1",(double\*)0);

102  stan::math::check\_vector("dot\_product(%1%)",v2,"v2",(double\*)0);

103

104  fvar<T> ret(0,0);

105  for(size\_type i = 0; i < length; i++)

106  ret += v1(i) \* v2(i);

107  return ret;

108  }

109

110  template<typename T>

111  inline

112  fvar<T>

113  dot\_product(const std::vector<fvar<T> >& v1,

114  const std::vector<fvar<T> >& v2) {

115  stan::math::check\_matching\_sizes("dot\_product(%1%)",v1,"v1",

116  v2,"v2",(double\*)0);

117  fvar<T> ret(0,0);

118  for (size\_t i = 0; i < v1.size(); i++)

119  ret += v1.at(i) \* v2.at(i);

120  return ret;

121  }

122

123  template<typename T>

124  inline

125  fvar<T>

126  dot\_product(const std::vector<double>& v1,

127  const std::vector<fvar<T> >& v2) {

128  stan::math::check\_matching\_sizes("dot\_product(%1%)",v1,"v1",

129  v2,"v2",(double\*)0);

130  fvar<T> ret(0,0);

131  for (size\_t i = 0; i < v1.size(); i++)

132  ret += v1.at(i) \* v2.at(i);

133  return ret;

134  }

135

136  template<typename T>

137  inline

138  fvar<T>

139  dot\_product(const std::vector<fvar<T> >& v1,

140  const std::vector<double>& v2) {

141  stan::math::check\_matching\_sizes("dot\_product(%1%)",v1,"v1",

142  v2,"v2",(double\*)0);

143  fvar<T> ret(0,0);

144  for (size\_t i = 0; i < v1.size(); i++)

145  ret += v1.at(i) \* v2.at(i);

146  return ret;

147  }

148

149  template<typename T>

150  inline

151  fvar<T>

152  dot\_product(const std::vector<fvar<T> >& v1,

153  const std::vector<fvar<T> >& v2,

154  size\_type& length) {

155  fvar<T> ret(0,0);

156  for(size\_type i = 0; i < length; i++)

157  ret += v1.at(i) \* v2.at(i);

158  return ret;

159  }

160

161  template<typename T>

162  inline

163  fvar<T>

164  dot\_product(const std::vector<double>& v1,

165  const std::vector<fvar<T> >& v2,

166  size\_type& length) {

167  fvar<T> ret(0,0);

168  for(size\_type i = 0; i < length; i++)

169  ret += v1.at(i) \* v2.at(i);

170  return ret;

171  }

172

173  template<typename T>

174  inline

175  fvar<T>

176  dot\_product(const std::vector<fvar<T> >& v1,

177  const std::vector<double>& v2,

178  size\_type& length) {

179  fvar<T> ret(0,0);

180  for(size\_type i = 0; i < length; i++)

181  ret += v1.at(i) \* v2.at(i);

182  return ret;

183  }

184  }

185 }

186 #endif

typedefs.hpp

stan::agrad::fvar

**Definition:** fvar.hpp:13

fwd.hpp

typedefs.hpp

stan::length

size\_t length(const T &)

**Definition:** traits.hpp:159

stan::math::check\_vector

bool check\_vector(const char \*function, const Eigen::Matrix< T, R, C > &x, const char \*name, T\_result \*result)

**Definition:** check\_vector.hpp:14

check\_matching\_sizes.hpp

stan::agrad::size\_type

Eigen::Matrix< double, Eigen::Dynamic, Eigen::Dynamic >::Index size\_type

**Definition:** typedefs.hpp:14

stan::agrad::dot\_product

fvar< T > dot\_product(const Eigen::Matrix< fvar< T >, R1, C1 > &v1, const Eigen::Matrix< fvar< T >, R2, C2 > &v2)

**Definition:** dot\_product.hpp:20

Eigen.hpp

to\_fvar.hpp

check\_vector.hpp

stan::math::check\_matching\_sizes

bool check\_matching\_sizes(const char \*function, const T\_y1 &y1, const char \*name1, const T\_y2 &y2, const char \*name2, T\_result \*result)

**Definition:** check\_matching\_sizes.hpp:15


---

[ Stan Home Page ]

*© 2011–2014,
Stan Development Team.*
